# Serous endometrial intraepithelial carcinoma: a case report

**DOI:** 10.11604/pamj.2023.44.122.37712

**Published:** 2023-03-10

**Authors:** Younesse Najioui, Nassia Karich, Anas Haloui, Achraf Miry, Amal Bennani

**Affiliations:** 1 Department of Pathology, Mohammed VI University Hospital, Faculty of Medicine and Pharmacy of Oujda, Mohammed First University of Oujda, Oujda, Morocco

**Keywords:** Serous endometrial intraepithelial carcinoma, serous carcinoma, endometrium, case report

## Abstract

Serous endometrial intraepithelial carcinoma (SEIC) is a rare but highly aggressive form of uterine endometrial cancer. We report two cases of post-menopausal, 58-year-old patients with abundant vaginal bleeding and pelvic pain. The first patient had a history of surgical hysteroscopy in 2019 for an endocervical polyp. The second patient had a history of breast resection, axillary lymph node dissection, chemotherapy, radiation therapy, and tamoxifen therapy for breast carcinoma 6 years ago. An abdominal hysterectomy was performed in both patients. The pathological assessment showed serous endometrial intraepithelial carcinoma. Diagnosis of a serous proliferation of the uterus implies the exploration of other genital tract organs as well as distant locations in search of metastatic disease

## Introduction

Endometrial carcinoma is the most common malignant tumor of the female genital tract. Type 1 endometrial carcinoma is the most frequent type of endometrial carcinoma. It usually presents as a low-grade tumor with a favorable prognosis and a 5-year survival rate of reaching 85% [1]. Type endometrial carcinoma is an estrogen-independent type [2,3].

Serous endometrial intraepithelial carcinoma (SEIC) is the non-invasive precursor of invasive uterine serous carcinoma. In SEIC, a replacement of surface and/or glandular epithelium by neoplastic cells occurs, without invasion of the endometrial stroma. This condition is most often associated with endometrial atrophy or within an endometrial polyp [4]. Even if SEIC is confined to the endometrial mucosa, recurrences, and even deaths do occur [2,3]. Serous endometrial intraepithelial carcinoma is a difficult histopathological diagnosis without ancillary immunohistochemistry [5]. Molecular studies have clarified that SEIC is a potential source of distant metastatic disease, explaining why SEIC should be considered as an aggressive disease [6,7]. We report two cases of post-menopausal SEIC and will provide a literature review.

## Patient and observation

### Case 1

**Patient information:**a 58-year-old post-menopausal woman had been to our hospital. She suffered from abundant vaginal bleeding with pelvic pain. In 2019, she had a hysteroscopy for an endocervical polyp.

**Clinical findings:**the gynecological examination found no abnormalities.

**Diagnostic assessment:**the transvaginal ultrasound identified an endometrial thickening of 17 mm, suggestive of an intracavitary polyp. Hysterosonography detected an intracavitary polyp located in the anterior uterine wall. An endometrial curettage has been performed and showed during a pathological assessment a micropapillary and papillary proliferation of markedly atypical cells in favor of serous carcinoma.

**Therapeutic interventions:**for hemostasis, a total abdominal hysterectomy with bilateral adnexectomy was performed. A gross examination showed a normal endometrial mucosa, with the presence of a small round polyp arising from the uterine posterior wall.

**Figure 1:**
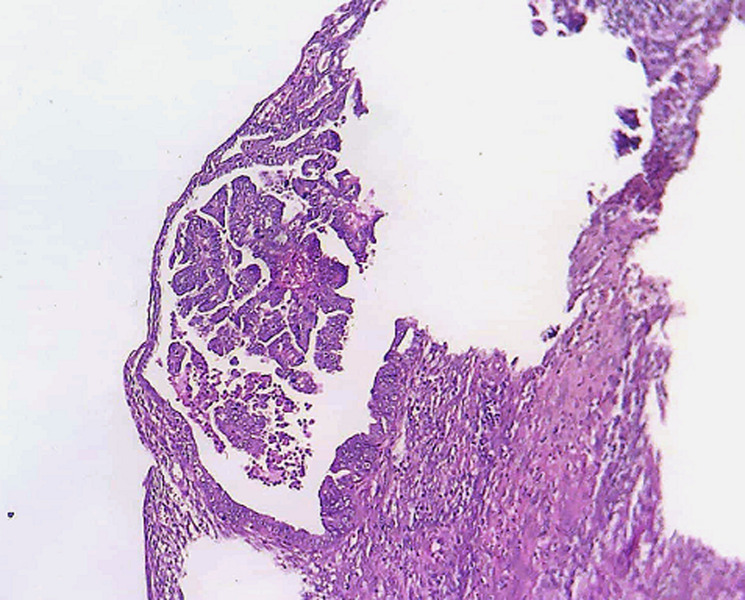
microphotography showing a proliferation of papillary and micropapillary architecture projecting from the endometrial surface, (hematoxylin and eosin, 100X)

**Figure 2:**
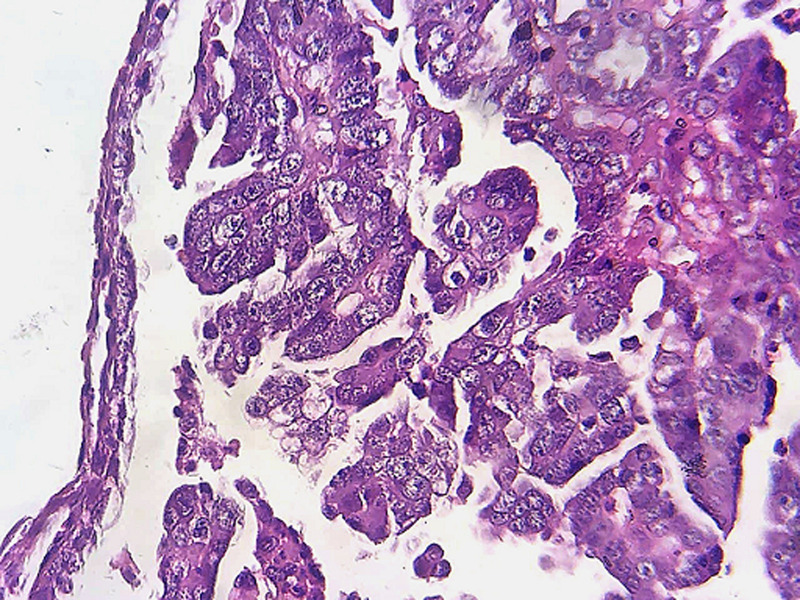
microphotograph of the observed micro-papillae, showing markedly atypical carcinomatous cells; these cells show a hobnail appearance; they have rounded or oval and nucleolate nuclei and an eosinophilic cytoplasm, (hematoxylin and eosin, 400X)

**Follow-up and outcome of interventions:**histologically, samples taken from the uterine fundus revealed an atrophic endometrial mucosa, with the presence of a proliferation of papillary and micropapillary architecture projected on the surface. It measured less than 1 cm along its major axis. These micropapillary structures are bordered by atypical tumor cells with a hobnail appearance. The nuclei are rounded or oval, with a prominent nucleolus. Cytoplasm is eosinophilic. No invasion of the endometrial stroma has been identified. Furthermore, the presence of an endometrial polyp is noted (Figure 1 and Figure 2). The use of anti-ki-67 antibodies showed a high proliferation rate in the proliferation of neoplastic cells. After surgery, thoracic, abdominal, and pelvic computerized tomography (CT) was performed and showed no distant metastases. The multidisciplinary team decided to perform a bilateral salpingo-oophorectomy, omentectomy, and pelvic and para-aortic lymph node dissections. The pathological assessment found no invasive lesions and no infiltration of the ovaries, lymph nodes, or omentum. A final diagnosis of SEIC was established. Six months later, our patient presented umbilical cutaneous, pleural, and peritoneal metastases. The diagnosis was confirmed by a pathological assessment of skin and peritoneal carcinosis samples, performed during laparoscopy. The patient is currently under chemotherapy.

**Patient consent:**the patient's family has given informed consent about the publication of this work.

**Patient perspective:**the patient's family was pleased with the care she received throughout therapy.

### Case 2

**Patient information:**a 58-year-old menopausal female patient, presented to the gynecology obstetrics department for post-menopausal vaginal bleeding. She had a history of diabetes treated with oral anti-diabetics for a period of 8 years. She also had a history of breast carcinoma for which she has undergone breast resection with lymph node dissection, chemotherapy, radiation therapy, and tamoxifen intake 6 years ago.

**Clinical findings:**gynecological examination didn´t find any bleeding from the cervical Os during speculum examination.

**Diagnostic assessment:**pelvic ultrasounds revealed a suspicious thickened endometrium with an interstitial lesion suggesting an anterior 41x42mm myoma. A thoracic, abdominal, and pelvic CT scan has been also performed revealing no metastatic locations. The patient has then undergone hysteroscopy with endometrial curettage. The samples were received in our department and microscopic examination revealed an intra-epithelial proliferation of malignant atypical cells with marked nuclear atypia in form of nuclear enlargement, hyperchromasia, and frequent mitoses. The cytoplasm was eosinophilic. These findings, along with the absence of stromal invasion were consistent with intra-epithelial serous carcinoma of the endometrium.

**Therapeutic interventions:**a total hysterectomy, bilateral salpingo-oophorectomy, and bilateral iliac lymph nodes dissection were performed.

**Figure 3:**
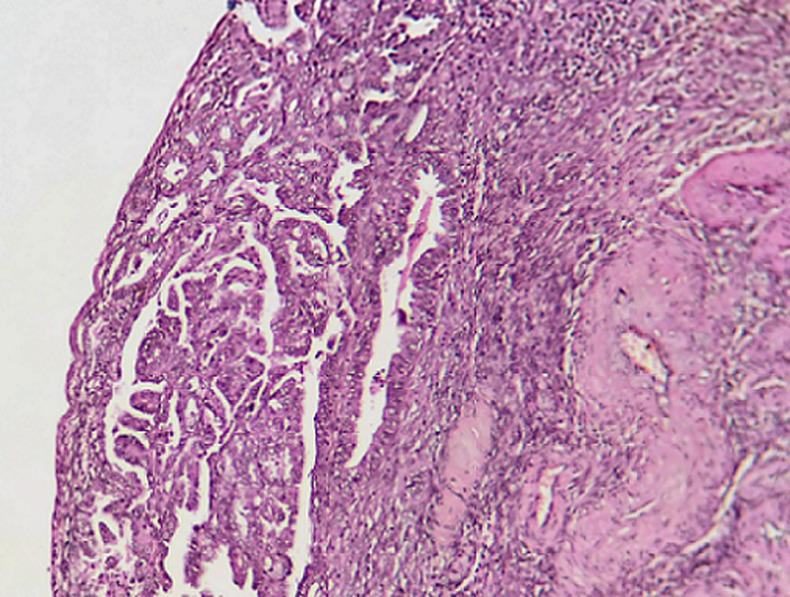
microphotography showing presence of an intra-epithelial proliferation of malignant atypical cells, (hematoxylin and eosin, 200X)

**Figure 4:**
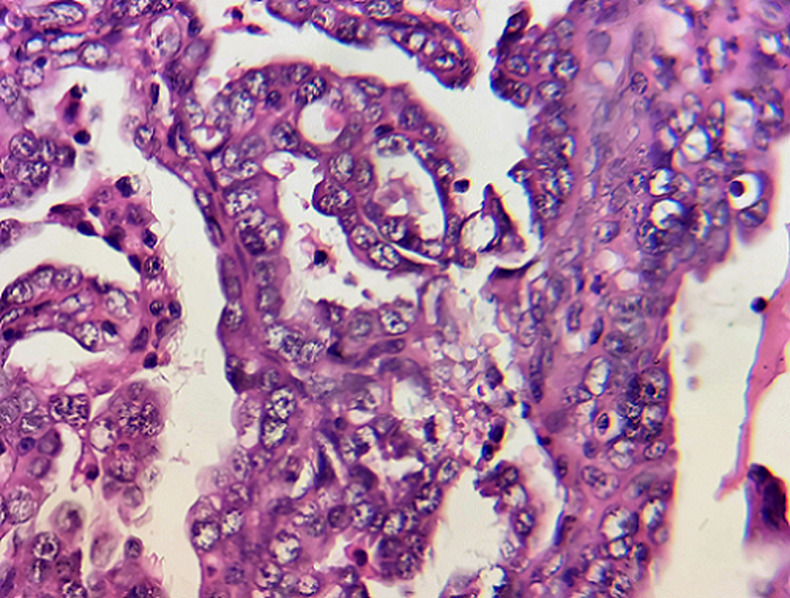
microphotography at higher magnification showing nuclear enlargement and hyperchromasia; the cytoplasm is eosinophilic; these findings, along with absence of stromal invasion are consistent with intra-epithelial serous carcinoma of the endometrium, (hematoxylin and eosin, 400X)

**Follow-up and outcome of interventions:**microscopic assessment revealed the same intra-epithelial proliferation as shown in the curettage samples. The neoplastic cells were proliferating in form of cystic invagination into the endometrial stroma but constantly respecting a basal membrane between the tumor cells and the endometrial stroma. These cysts contained papillae and micropapillary structures. The neoplastic cells often showed a hobnail morphology. No invasion of the endometrial stroma was observed. All lymph nodes were free of neoplastic proliferation (Figure 3 and Figure 4). Follow-up revealed no metastatic lesions after a period of 7 months.

**Patient consent:**the patient's family has given informed consent about the publication of this work.

**Patient perspective:**the patient's family was pleased with the care she received throughout therapy.

## Discussion

Serous endometrial intraepithelial carcinoma has been regarded as an early form of serous endometrial carcinoma (SEC). One of the relevant proofs for this theory is the presence of the same mutations in both SEIC and SEC components when they are observed in a combined fashion [8]. Serous endometrial intraepithelial carcinoma shares the epidemiological and clinical features of serous endometrial carcinoma. They both occur most often in multiparous postmenopausal women. Current smoking, tubal ligation, history of breast cancer (as in our second reported case), and tamoxifen intake are risk factors. Obesity is less observed in cases of serous carcinoma and serous intra-epithelial carcinoma when compared to cases of endometrioid carcinoma.

Clinically, both serous carcinoma and serous intra-epithelial carcinoma present in form of postmenopausal bleeding. The clinical examination is classically poor and finds no relevant clues for the diagnosis [4]. Radiological exploration through CT-scan, MR imaging, or hysteroscopy most often shows an endometrial thickening [4]. On microscopic examination, cytological features of proliferating cells in cases of serous intra-epithelial carcinoma are the same as observed in cases of invasive serous endometrial carcinoma. Atypia is marked, nuclei are enlarged, atypical, pleomorphic, and show high mitotic rates [4]. On the histochemical level, the most relevant used antibody remains to be the anti-p53 antibody which is found to be overexpressed in nearly all cases of SEIC. The use of anti-ki-67 antibodies reveals the high proliferation rate as already shown by the presence of numerous mitotic figures [9].

The differential diagnosis should include an early form of invasive serous endometrial carcinoma. Although no strict criteria have been established to differentiate the two entities, some clues should be in favor of the diagnosis of invasion, namely the confluent glandular pattern, stromal desmoplasia, and a dimension larger than 1cm. importantly, many described cases have shown metastatic sites of serous carcinoma without evidence of in-stromal invasion on uterine samples [9].

## Conclusion

Serous endometrial intraepithelial carcinoma (SEIC) is a rare but highly aggressive form of uterine endometrial cancer. Molecular studies have clarified that SEIC is a potential source of distant metastatic disease, explaining why SEIC should be considered as an aggressive disease. Diagnosis of a serous proliferation of the uterus imply exploration of other genital tract organs as well as distant locations in search for a metastatic disease.
